# Arthritis Treated by Iodine-Lithium Ionisation

**Published:** 1909-09-18

**Authors:** 


### ARTHRITIS TREATED BY IODINE-LITHIUM IONISATION.

An interesting case of the relief of traumatic arthritis by iodine-lithium ionisation was recently recorded by Dr. Finzi before the Royal Society of Medicine. The patient, a policeman, aged 29, was kicked on the left knee on May 8, 1907. An attack of synovitis followed, which subsided, but as soon as the patient started to work again, in August 1907, another attack occurred. He was admitted to the Metropolitan Hospital, and by means of massage and hot-air baths was sufficiently improved to resume work in October 1907. The knee then gradually became worse, and from time to time was the seat of great pain. In July 1908 the patient was forced to give up work again, and from July 20 to August 5 he was treated with the high-frequency current with slight improvement. At this time the tissues around the knee were very much thickened, the synovial membrane being felt to be very thick; there was considerable grating in the joint, and the patient was unable to walk without the help of a stick. Skiagrams showed very little bony change in the knee, but definite osteo-arthritic changes in the hands. From August 7 to September 21 the patient was treated with iodine-lithium ionisation. A thick pad of cotton-wool soaked in lithium citrate, covered with a metallic electrode and attached to the positive pole of a galvanic current, was placed on one side of the knee, and a similar pad soaked in potassium iodide, connected to the negative pole, on the other side. These were firmly bandaged on, and the current was increased gradually to about 70 ma., and allowed to pass for 45 minutes. This soon relieved the pain and other symptoms. Before the treatment was stopped the patient walked 30 miles, and has subsequently come in second in a running race. By September 21, 1908, there was great diminution in the thickening around the knee, and the joint now appeared to be normal.

